# Comparison of sublingual microcirculatory parameters measured by sidestream darkfield videomicroscopy in anesthetized pigs and adult humans

**DOI:** 10.1002/ame2.12348

**Published:** 2023-09-03

**Authors:** Raushan Lala, Ryan Homes, Shaun Pratt, Wendy Goodwin, Mark Midwinter

**Affiliations:** ^1^ School of Biomedical Sciences The University of Queensland St Lucia Qld Australia; ^2^ School of Veterinary Sciences The University of Queensland Gatton Qld Australia; ^3^ Jamieson Trauma Institute, Royal Brisbane and Women's Hospital Herston Qld Australia; ^4^ Traumatic Injury Sciences Group The University of Queensland St Lucia Qld Australia

**Keywords:** comparative anatomy, microcirculation, sidestream darkfield videomicroscopy

## Abstract

**Background:**

This study aimed to compare sublingual microcirculatory parameters between anesthetized pigs and conscious adult humans using sidestream darkfield videomicroscopy. The overarching aim of the work was to validate the pig as an experimental model of changes in microcirculatory function following traumatic haemorrhagic shock and resuscitation.

**Methods:**

Fourteen large white pigs and 14 humans were recruited for the study. Sublingual sidestream darkfield videomicroscopy clips were captured in anesthetized pigs and conscious humans. Clips underwent manual analysis in Automated Vascular Analysis 3.2 software. The total vessel density (TVD), perfused vessel density (PVD), proportion of perfused vessels (PPVs) and microvascular flow index (MFI) were quantified. An independent samples t test was used for between species comparison of microcirculatory parameters.

**Results and Conclusions:**

Conscious humans had a significantly lower TVD, PVD and MFI than anesthetized pigs. No significant difference in PPVs was observed between the species. Perfusion of the microcirculation is a critical determinant of tissue metabolic function and viability. Whilst it may not be surprising that some *inter*species differences in the sublingual microcirculatory anatomy were identified between pig and human subjects, it is interesting to report the insignificant difference in PPVs. This direct microcirculatory measure represents a relative change which should hold translatable value across species. We therefore conclude the pig is a suitable model for microcirculatory research and may be a suitable species to investigate changes in microcirculatory perfusion following perturbations in cardiovascular homeostasis, for example during traumatic haemorrhagic shock and resuscitation.

## Graphical abstract

Pigs are commonly used to model human microcirculatory function in various clinical states. We have compared the sublingual microcirculation in anesthetized pigs and conscious humans using sidestream darkfield videomicroscopy to validate the pig as a suitable model organism. Pigs had a higher total vessel density than humans, indicating an interspecies difference in microcirculatory anatomy. The proportion of perfused vessels (critical measure for tissue perfusion) however was not significantly different between the species (functional similarity).

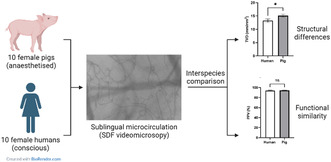

## 1 INTRODUCTION

Traumatic injury is a significant contributor to global mortality and remains the leading cause of death in patients under 41 years.^1^ In patients surviving initial injuries, development of multiple organ dysfunction syndrome (MODS) contributes to later mortality.^2^ The MICROSHOCK study demonstrated an association between early microcirculatory dysfunction and development of MODS in trauma patients 1 week after injury.^3^ This work motivated significant research effort towards defining the role of microcirculatory function in pathophysiologic responses to traumatic injury, haemorrhagic shock and subsequent resuscitation.

The microcirculation is the part of the circulatory system where metabolic exchange between blood and tissues occurs. Adequate microcirculatory perfusion requires sufficient vessel density and conductance of oxygenated red blood cells, with hypoperfusion resulting from compromise to either of these parameters. Vessel density and microcirculatory perfusion can be evaluated by sidestream darkfield (SDF) videomicroscopy, for which analysis protocols have been developed by expert consensus.^4^

Population heterogeneity in trauma cohort studies relating to comorbidities, injury mechanisms, prehospital care and timing of interventions complicate the study of pathophysiologic changes in microcirculatory function. Animal models have therefore been used to standardize many of these parameters and allow for the examination of microcirculatory responses to traumatic haemorrhagic shock in a controlled environment. Rodent models, despite husbandry and cost benefits, are of limited use in trauma research due to their low blood volumes (precluding sequential sampling, especially on a background of hemorrhage) and clinically relevant anatomical differences compared to humans. Larger animal species, such as pigs, address these issues and are therefore among the most frequently reported large animal trauma model.^5^

Our research group has developed a porcine model of severe traumatic injury and haemorrhagic shock to investigate the temporal profiles of microcirculatory (dys)function following injury, shock and resuscitation. Various groups have conducted sublingual videomicroscopy in pigs or humans independently; however, a direct comparison between the species has not been reported. An investigation into the potential baseline differences between model species is essential if relevant conclusions are to be translated or used as a basis for development of human studies. This work therefore aimed to compare the sublingual microcirculation in a population of healthy human adults to that of healthy, anesthetized pigs.

## 2 METHODS

### 2.1 Experimental design

This study was undertaken with ethical approval from both human and animal ethics committees (HREC: 2021/HE001851 and AEC: 2021/AE000830). Sublingual videomicroscopy was undertaken in 14 healthy adult humans and 14 healthy, anesthetized pigs.

### 2.2 Porcine anesthesia protocol

Pigs were sedated with 2 mg/kg azaperone (Stresnil, Elanco Australasia Pty Ltd) intramuscular. Masked oxygen was provided while a 22G 1 inch catheter (Surflo, Terumo) was placed into an auricular vein. General anesthesia was induced 5 min later with preservative‐free alfaxalone (Alfaxan, Jurox) intravenous (IV) titrated slowly to effect. The larynx was desensitized topically with 40 mg lidocaine 2% (Lignocaine 20, Troy Laboratories) prior to intubation with an appropriately sized polyvinyl chloride, cuffed orotracheal tube and connection to the re‐breathing circuit of a Mindray WATO anesthetic machine (Shenzhen Mindray Bio‐Medical Electronic Co., Ltd). Partial IV anesthesia was maintained using alfaxalone (4 mg/kg/h), midazolam (Hypnovel, Roche Products; 0.4 mg/kg/h) and isoflurane (Isofol, Zoetis) delivered in a mixture of 30% enriched oxygen and 70% medical air. Pressure controlled mechanical ventilation was set to deliver peak inspiratory pressures of 10–15 mm Hg, an inspiratory time of 1.1–1.3 s and a respiratory rate adjustable to maintain end‐tidal normocapnia (45–55 mm Hg).

### 2.3 Sublingual microcirculatory imaging

Humans and anesthetized pigs were both positioned in dorsal recumbency. Human participants were placed on a physiotherapy plinth with the backrest angle set to 40°, whereas pigs were supine. Five sublingual microcirculatory clips were captured using SDF videomicroscopy (MicroScan, MicroVision Medical). Analysis of microcirculatory clips was undertaken using Automated Vascular Analysis 3.2 (AVA; Academic Medical Centre, University of Amsterdam). Clip quality was appraised using the Microcirculatory Image Quality Score (MIQS) and only clips with MIQS <10 were included for analysis. The following parameters were generated by the analysis: total vessel density (TVD), perfused vessel density (PVD), proportion of perfused vessels (PPV) and microvascular flow index (MFI). The computation of the above parameters has been described elsewhere.^4^ Briefly, all vessels were traced manually and gridlines were superimposed over the image. The TVD was derived from the number of intersections between the traced vessels and gridlines. The flow of red blood cells through each vessel was then rated on an ordinal scale of 0–3 by direct visualization (0 = no flow; 1 = intermittent flow; 2 = sluggish flow; 3 = continuous flow). The PPV was the proportion of total vessels with a rating of sluggish or continuous flow expressed as a percentage of the TVD, and the PVD calculated by multiplying the TVD by PPV. Calibration settings used in this study are attached in File S1. The parameters were computed and reported in accordance with consensus guidelines for examination of microcirculatory function.^4^ All microcirculatory clip analysis was undertaken by a trained operator blinded to the species origin of the clips (RL).

### 2.4 Statistical analysis

Normality was confirmed for microcirculatory data by the Shapiro–Wilk test and mean values for each microcirculatory parameter were compared using an independent samples *t* test. Microcirculatory data are presented as mean and standard error, and demographic data are presented as median and interquartile range. The statistical significance threshold was set to *p* < 0.05. A correction for multiple *t* tests was not undertaken. All statistical analysis and graphing was undertaken in GraphPad Prism 9.4.1 (GraphPad Software).

## 3 RESULTS

### 3.1 Subject demographics

Microcirculatory assessment was performed in 14 pigs and 14 humans—all subjects were healthy, non‐pregnant females. Large white pigs were anesthetized for the study whereas human participants were conscious. Demographics for each group are presented in Table 1.

**TABLE 1:** Study demographics.

	Pig	Human
*n*	14	14
Sex (F:M)	14:0	14:0
Age	12 weeks	57 (24.5–76) years
Weight (kg)	34.75 (21–36)	60.5 (55.35–75.75)
Breed	Large white	—

*Note*: Data are presented as median and interquartile range.

### 3.2 Comparison of microcirculatory parameters

**FIGURE 1:**
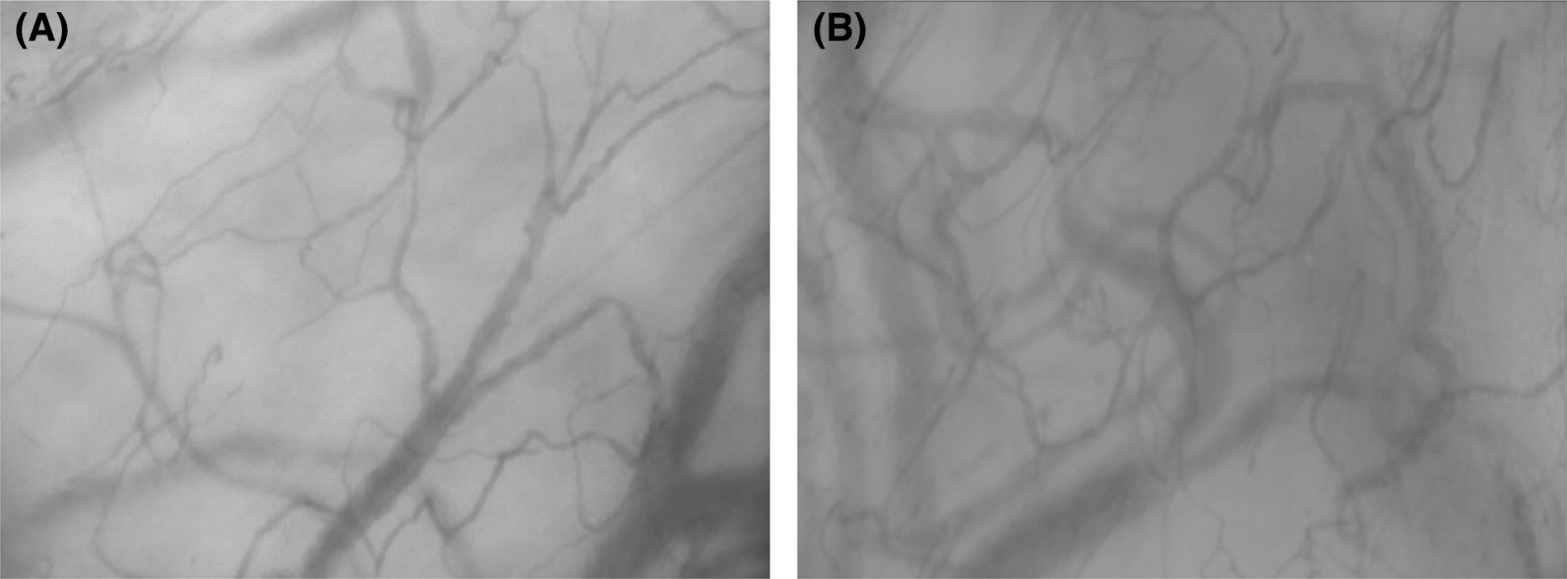
Example frame from sublingual videomicroscopy clip of human (A) and porcine (B) sublingual microcirculation. Though these images provide some indication of vessel density, single frames provide no information regarding vessel perfusion status (flow metrics analyzed across a series of frames).

Microcirculatory parameters were calculated after manual vessel segmentation. Representative examples of the human and porcine sublingual microcirculation are displayed in Figure 1. Example microcirculatory clips (from which the still images in Figure 1 were derived) are available in File S2.

**FIGURE 2:**
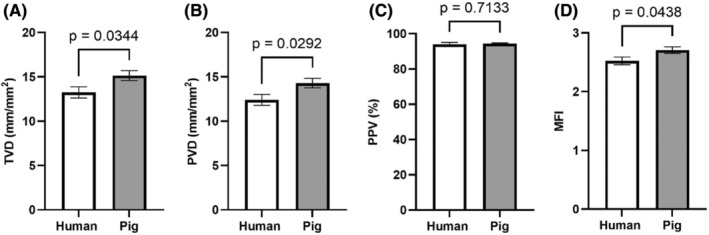
Sublingual microcirculatory parameters in pigs and humans. Data are presented as mean ± SE. (A) TVD, total vessel density; (B) PVD, perfused vessel density; (C) PPV, proportion of perfused vessels; (D) MFI, microvascular flow index.

Comparisons between the species were made for TVD, PVD, PPV and MFI. In comparison to humans, pigs had a significantly higher TVD (pig 15.15 ± 0.56; human 13.25 ± 0.64; *p* = 0.034), PVD (pig 14.29 ± 2.03; human 12.41 ± 0.61; *p* = 0.029), and MFI (pig 2.71 ± 0.06; human 2.52 ± 0.06; *p* = 0.044; Figure 2A,B,D). However, no significant difference in PPV was observed (pig 94.40 ± 0.43; human 93.92 ± 1.23; *p* = 0.071; Figure 2C).

## 4 DISCUSSION

This study compared the sublingual microcirculation of anesthetized pigs to human adults. Our research group has developed a porcine model of traumatic haemorrhagic shock to investigate the temporal profiles of microcirculatory function after injury. The current study was conducted to validate the pig as a suitable model of human microcirculatory function. To our knowledge, such a species comparison has not been undertaken.

Anesthetized pigs displayed higher vessel densities and microvascular flow indices than humans; however, no species difference in PPV was observed. Although no previous studies directly compare species, several studies involving either pigs or humans have reported baseline microcirculatory parameters. Previously reported PVD values vary for healthy adult humans, ranging from approximately 8 mm/mm^2^ ^6^ to 25.4 mm/mm^2^.^7^ The cause of marked variability between these studies is unclear, as both human populations were of comparable demographics, and image acquisition and analysis followed similar protocols, though software calibration settings were not reported. Previous work involving anesthetized pigs report a PVD at baseline of approximately 14 mm/mm^2^ (value extrapolated from published figure),^8^ which is comparable to values reported in the present study (within the reported range for human PVD).

The difference in TVD between the pig and human may be due to inter‐species anatomical variation. Differences in macrovascular anatomy of the carotid arterial system between the species have been demonstrated radiographically, which may translate to the associated microvessels.^9^ Importantly, however, the PPV in the sublingual microcirculatory beds did not differ between species. We propose, that *within* a species, PPV is the most relevant parameter to reflect changes in the microcirculatory bed because of perturbations in cardiovascular homeostasis, such as hemorrhage and resuscitation. Given sufficient vessel density to supply the extent of a tissue block (appropriate density may vary between species), it is the perfusion status of the vessels which define the function of the microcirculation. On this basis we propose PPV is the most relevant parameter when examining tissue perfusion responses to acute changes in cardiovascular hemostasis. This is supported by previous (small) trauma cohort studies wherein PPV showed greater predictive value than other microcirculatory parameters for the development of MODS.^10^ It should be noted, however, that this finding has not been validated in larger trauma cohorts.^3^

The comparison of microcirculatory parameters at baseline (i.e. prior to injury and hemorrhage) to validate a trauma model may have limitations. The validity of the model may be more dependent on the similarity of the response to traumatic injury between the pig and human microcirculation, rather than simply the pre‐injury parameters. In this case, the concordance of microcirculatory parameters between the species at baseline is limited in validating the pig as a human microcirculatory model; however, comparison of responses cardiovascular perturbations (particularly traumatic haemorrhagic shock) under laboratory conditions is not feasible.

Due to ethical and logistical issues, all pigs were anesthetized for videomicroscopy. This could be responsible for some of the observed microcirculatory differences and limits the validity of the study as a direct species comparison under non‐experimental conditions. The anesthetic protocol was designed to administer agents which minimally alter microcirculatory function. For example, isoflurane has previously been shown to minimally impact microcirculatory parameters in humans.^11^

Supplemental oxygen was provided during anesthesia in the current study and may have impacted the microcirculatory parameters reported. The relationship between fraction of inspired oxygen (FiO_2_) and sublingual microcirculatory function under general anesthesia remains to be investigated; however, in conscious humans, a shift from room air to 30% FiO_2_ delivered by a non‐invasive ventilation mask does not alter the sublingual microcirculation.^12^ We therefore propose that although the role of anesthesia is an important consideration (particularly where the modeled clinical entity occurs in conscious adult humans), there is limited evidence for a confounding effect of the anesthetic protocol used in this study. Furthermore, since the primary aim was to assess the validity of the pig as an experimental model of microcirculatory responses to acute traumatic injury and hemorrhage, we believe the most appropriate approach to validate model was to compare the microcirculation of anesthetized pigs to conscious humans.

The significant age disparity between the pigs and human participants is a potential confounding factor. The effect of age on the microcirculation has not yet been elucidated, nor has the developmental trajectory of the sublingual microcirculation. At the time of writing, there is an ongoing observational study to this end (NIH identifier: NCT05324228) which may provide further insight into this potential variable.

All participants involved in this study were female owing to the design of the larger porcine trauma study. There is well documented sexual dimorphism regarding cardiovascular responses to traumatic haemorrhagic shock which has been reviewed elsewhere.^13^ We therefore included only female animals in the trauma study and matched this with enrolled human participants. Though it would be of interest to repeat the current study with male participants, previous work has demonstrated no significant difference in any microcirculatory parameters between human males and females.^14^

The postural difference between the humans (supine with backrest set to 40°) and the pigs (supine) may impact the microcirculation. In humans, maximizing image quality was the primary consideration when determining backrest position. We found the sublingual mucosa to be most accessible to the SDF videomicroscope with the backrest elevated. It should also be noted this work was conducted in conscious adults and hence their comfort was also considered. The animals in our study, as with most trauma models, were supine for reasons relating to the associated trauma experiment. Optimisation of patient positioning for SDF image acquisition has not yet been reported in the literature. However, Klijn et al.,^15^ reported that, in people, postural change from 70° head up tilt to supine, does not significantly alter sublingual microcirculatory parameters. It should also be noted that the normal anatomic position of the pig and human differs, with pigs standing upright on the phalanges of each fore and hindleg (unguligrade stance) compared with humans who stand upright in a plantigrade stance. It is unknown if these normal postural differences result in differences between the sublingual microcirculation.

Several future directions have become evident from the current work. A more robust characterization of the effects of various anesthetic protocols on the microcirculation is needed. A cohort study of this nature could be conducted in surgical patients with measurements being collected before induction, following induction prior to incision, and then at set time points during surgery. Regarding comparative anatomy, several other large animal species are commonly employed in translational research (sheep, dogs, primates). Inter‐species comparisons would be warranted in particular to examine if the PPV is preserved to maintain tissue viability across a range of species.

Further studies are required to examine the reliability of the sublingual microcirculation as a marker of microcirculatory function of other vascular beds. This will be achieved in future studies involving the current porcine model wherein SDF imaging of the colonic mucosa will be undertaken via a loop stoma externalized through a lateral abdominal trephine in addition to standard sublingual imaging.

This model could be adapted for alternative areas of research in critical illness. The effect of vascular haemostatic interventions after injury including resuscitative balloon occlusion of the aorta on microcirculatory function has yet to be characterized. Work in this field was recently reported; however, the microcirculation was examined by tissue oxygenation measures rather than videomicrosopic visualization.^16^ Beyond trauma, a model similar to that presented here would have utility in sepsis research. Pigs are already commonly used to model sepsis with multiple recent studies examining sublingual microcirculatory function in this context.^17^, ^18^ The validation data presented here is supplementary to the existing body of work involving use of pigs to model critical illness and informs researchers adopting a porcine model to study microcirculatory changes in disease of some differences between pig and human but the similarity in sublingual PPV.

## AUTHOR CONTRIBUTIONS

Raushan Lala: conceptualisation, data acquisition, data analysis, writing—original draft, writing—reviewing and editing. Ryan Homes: conceptualisation, data acquisition, data analysis, writing—original draft, writing—reviewing and editing. Shaun Pratt: conceptualisation, data acquisition, writing—reviewing and editing. Wendy Goodwin: conceptualisation, funding acquisition, writing—reviewing and editing. Mark Midwinter: conceptualisation, funding acquisition, writing—reviewing and editing.

## FUNDING INFORMATION

Experimental work was funded by UQ Midwinter Group funds. RL received PhD candidature funding from the Australian Government Research Training Program.

## CONFLICT OF INTEREST STATEMENT

No conflicts of interests are declared by any author.

## ETHICS STATEMENT

2021/HE001851 (human ethics), 2021/AE000830 (animal ethics).

## Supporting information


File S1.

Data S1.

## Data Availability

Raw data will be made available upon request.
