# Supplementary material for: Comparison of sublingual microcirculatory parameters measured by sidestream darkfield videomicroscopy in anesthetized pigs and adult humans
Source: Animal Model Exp Med. 2023 Sep 3;6(5):499–503. doi: 10.1002/ame2.12348 (PMC10614120; doi:10.1002/ame2.12348)

**Supplementary Materials**

Supplement S1: Screenshot of calibration settings used in Automated Vascular Analysis (AVA) 3.2 software.


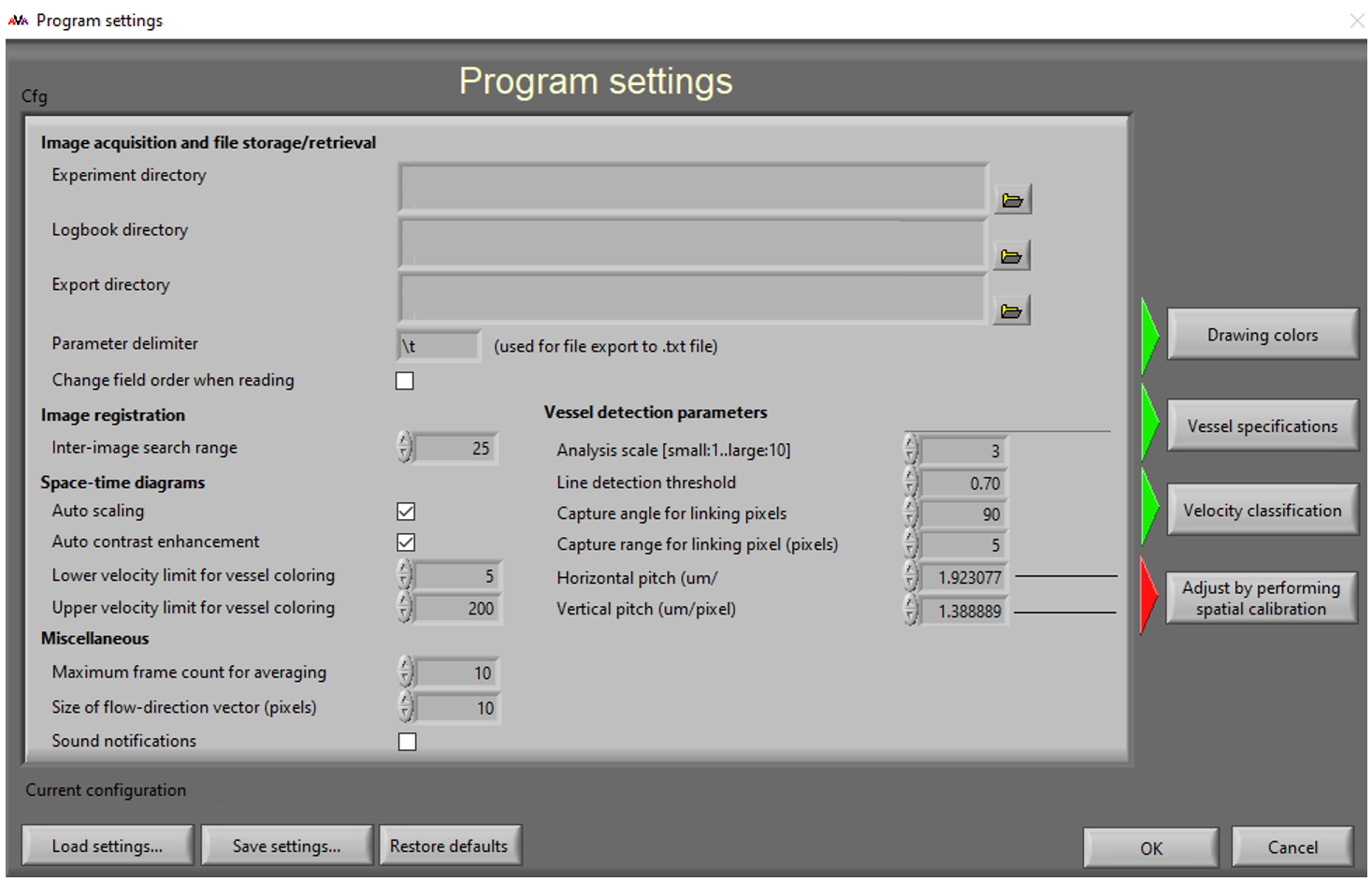

Supplement: Supplementary file 1 — File S1. [file AME2-6-499-s002.docx]
